# The I-95 Complication Corridor: High-Volume Cosmetic Surgery, Regulatory Blind Spots, and the Patients Left Behind

**DOI:** 10.7759/cureus.113146

**Published:** 2026-07-22

**Authors:** Andrew M Klapper, Anthony N Dardano, Michael Risin, Monali Mahedia

**Affiliations:** 1 Plastic and Reconstructive Surgery, Delray Medical Center, Delray Beach, USA

**Keywords:** adverse-event reporting, cosmetic surgery, florida, healthcare quality and safety, medical ethics, office-based surgery, patient safety, postoperative complications, public health, regulatory oversight

## Abstract

South Florida has become a major destination for affordable cosmetic surgery. Affordability is not the problem. This editorial argues that a recurring high-volume care-delivery model, characterized by aggressive marketing, financing-driven patient acquisition, unclear facility ownership, parallel operating rooms, nominal surgeon supervision, and weak postoperative accountability, may expose medically vulnerable patients to preventable harm and shift the consequences onto emergency departments, hospitals, public payers, families, and taxpayers. The central concern is not simply that complications occur. It is whether Florida’s reporting and oversight systems can connect downstream rescue care to originating facilities, surgeons of record, ownership structures, and repeat-pattern operators. This editorial proposes mandatory bidirectional adverse-event reporting, volume-triggered financial responsibility, procedure-specific privileging requirements, limits on simultaneous rooms under one surgeon of record, and enhanced ownership transparency. This is not a complication problem. It is an accountability and surveillance problem.

## Editorial

Opening vignette

The billboard did not advertise risk. It advertised transformation, a new body, a lower price, with financing available. The patient saw what thousands of patients have seen along the South Florida cosmetic surgery corridor: beauty presented as accessible, efficient, and financially within reach.

She had been told she would be watched after surgery. That promise helped make the decision feel safe. But "overnight monitoring" became something less definite after the operation ended. She was discharged quickly, still pale, still medicated, still in pain, and handed to people who could not recognize what postoperative hemorrhage, pulmonary embolism, sepsis, or evolving tissue loss might look like. The facility had completed the transaction. The patient had not completed the risk.

By the time she reached a hospital far from the original facility, the emergency department was no longer seeing an elective cosmetic surgery patient. It was receiving a surgical emergency. These patients do not arrive as abstract complications. They arrive tachycardic, hypotensive, anemic, septic, hypoxic, or in extremis. Some need blood before the operative records can be found. Some need broad-spectrum antibiotics, vasopressors, anticoagulation, intubation, interventional radiology, urgent debridement, or ICU admission before anyone has confirmed who truly owned the original operation. The question is not whether complications occur. They do. The question is whether the system was designed to own them.

The chart said Miami. The surgeon of record was difficult to reach. The facility had no meaningful ownership of the complication. The hospital inherited the patient. The reconstructive surgeon inherited the wound. The family inherited the fear. The payer inherited the bill.

This is the I-95 complication corridor. It is not an indictment of Miami, nor an attack on the many board-certified plastic surgeons who practice with judgment, continuity, and accountability. It is an indictment of a specific care-delivery model: high-volume, low-accountability cosmetic surgery facilities that sell the operation locally and export the complication regionally.

The surveillance gap: Florida cannot manage what it does not measure

Florida already has an adverse-event reporting framework. Section 458.351, Florida Statutes, requires reporting of specified adverse incidents in physician office settings, including death and "any condition that required the transfer of a patient to a hospital," within 15 days [1]. The state has already accepted the premise that office-surgery complications are not purely private events.

Published Florida data confirm a measurable burden. Coldiron et al., analyzing seven years of Florida adverse-incident reports, documented 31 deaths and 143 procedure-related complications and hospital transfers; liposuction, liposuction combined with abdominoplasty, and related cosmetic operations were heavily represented [2]. Starling et al. reported 309 Florida office-based adverse incidents over a 10-year period, including 46 deaths and 263 complications; cosmetic procedures accounted for 56.5% of deaths and 49.8% of hospital transfers [3]. South Florida BBL-specific literature documented 25 fat-embolism deaths between 2010 and 2022, with many cases associated with high-volume cosmetic surgery settings [4]. These studies predate or only partially capture the high-volume marketing and medical-tourism-style I-95 model; the current burden may be greater and less documented, not smaller.

The critical problem is not the absence of a reporting requirement. It is the absence of an integrated, publicly visible, bidirectional surveillance mechanism capable of linking downstream rescue care back to the originating cosmetic surgery facility, surgeon of record, ownership structure, volume model, financial responsibility status, and payer burden. A reporting requirement that captures isolated incidents but fails to identify repeat-pattern facilities is not surveillance. It is paperwork.

Florida's tragedy is not that no one at the bedside has been alarmed. Emergency physicians see it. Intensivists see it. Wound centers see it. Reconstructive surgeons see it. The state may not see the pattern because the pattern does not arrive in one piece. It arrives as a septic admission at one hospital, a transfusion at another, a pulmonary embolism at a third, an uncompensated-care write-off, a lawsuit, or a family that never files anything at all.

Clinicians see the corridor because they receive the downstream emergencies. The state misses the corridor because it receives only disconnected data points. Table 1 summarizes the evidence sources and surveillance gaps supporting this proposed bidirectional reporting framework.

**Table 1:** Evidence sources and surveillance gaps in Florida office-based cosmetic surgery This table summarizes the statutory and peer-reviewed sources supporting the proposed bidirectional reporting framework. It identifies what each source captures, what remains unlinked or unmeasured, and why Florida needs a surveillance pathway connecting downstream emergency rescue care back to originating cosmetic surgery facilities, surgeons of record, ownership structures, payer burden, and repeat-pattern operators

Evidence source	What it captures/what it misses/why bidirectional reporting is needed
Florida §458.351 adverse incident reporting statute [1]	Requires reporting of office-based adverse incidents, including death and hospital transfer, within 15 days. Does not appear to create a public, integrated mechanism linking downstream rescue care to the originating facility, surgeon of record, ownership structure, payer burden, or repeat-pattern operators
Coldiron et al., seven-year Florida adverse-incident data [2]	Documented deaths and complications/hospital transfers in Florida office surgery, with cosmetic procedures heavily represented. The dataset demonstrates measurable adverse-event burden but does not provide a current bidirectional linkage pathway from receiving hospitals back to originating facilities
Starling et al., Florida/Alabama office-surgery data [3]	Reported Florida office-based adverse incidents, including deaths and complications, with cosmetic procedures accounting for a substantial proportion of deaths and hospital transfers. The analysis does not provide public facility-level repeat-pattern tracking, ownership linkage, or payer-burden linkage
Pazmiño and Garcia, South Florida BBL mortality literature [4]	Documents geographically specific mortality associated with gluteal fat grafting in South Florida. It supports the relevance of the South Florida cosmetic surgery corridor but does not create a broader surveillance system for downstream complications across emergency departments, ICUs, and wound centers
Florida §458.320 financial responsibility statute [5]	Permits malpractice coverage, escrow, letter of credit, or non-insurance disclosure. It does not impose volume-adjusted financial responsibility on high-throughput elective cosmetic surgery facilities or link financial responsibility to downstream rescue burden

The high-volume care-delivery model and its structural risks

Business Architecture and Production Incentives

Cosmetic surgery can be performed safely in outpatient settings when patient selection, anesthesia, facility standards, and postoperative systems are real. Many board-certified plastic surgeons practice responsibly. Published Florida adverse-incident data and South Florida procedure-specific mortality data do not indict all outpatient cosmetic surgery; they identify a safety problem concentrated around high-risk procedures, inadequate systems, and high-volume settings [2-4]. Affordability is not the enemy of safety.

The problem is a care-delivery model that may industrialize risk. In its most concerning form, this model includes aggressive direct-to-consumer marketing, financing-driven patient acquisition, high daily case volume, out-of-town patient recruitment, unclear facility ownership, and physicians who may function more as regulatory faces than true owners of care. The surgical episode becomes a transaction rather than a relationship. The most dangerous part of the operation may be the business model.

Payment structure is often the clearest window into whether clinical judgment was independent or subordinated to production pressure. If a surgeon is compensated per case or by volume, the incentive favors throughput over cancellation. If a medical director receives a fixed fee to provide a name without real authority, the directorship may function as liability camouflage. If anesthesia providers are compensated in a way that rewards room turnover, the patient loses a critical independent safety check. A safe surgical system pays for judgment. A dangerous one pays for throughput and calls it access.

Multiple Rooms, One License, and the Borrowed License Problem

One of the central regulatory questions is whether a licensed surgeon can ethically, medically, or physically supervise multiple cosmetic surgery rooms simultaneously. If one surgeon is listed as responsible for several concurrent procedures, meaningful responsibility may be fictional. A license cannot supervise three rooms at once. A license is not a substitute for actual supervision, documented operative participation, lawful delegation, and direct accountability across parallel operating rooms.

A related concern is whether operative participation is accurately documented and lawfully supervised when multiple rooms are running under one surgeon of record. Foreign training is not the problem. The relevant patient-safety question is whether every individual materially participating in surgery is appropriately licensed, credentialed, supervised, and documented. If an individual performs liposuction, gluteal fat injection, abdominoplasty undermining, hemostasis, closure under tension, or intraoperative decision-making, that person is performing a critical surgical task, not merely assisting. Regulators should ask who performed each critical portion, what license and credentials that person held, whether the surgeon of record was physically present, and whether that surgeon was simultaneously responsible for another room. A surgeon’s name should not create the appearance of supervision where meaningful supervision, lawful delegation, and accurate operative documentation did not occur.

The Day-of-Surgery Stranger and the Monitoring Promise

In some high-volume facilities, patients are recruited, financed, scheduled, and medically "cleared" before the operating surgeon has ever met them. The surgeon may encounter the patient for the first time on the day of surgery, after travel, money, and expectations are already committed. A surgeon who first meets the patient after the operation has already been sold is not the beginning of the safety process. He is the last checkpoint in a sales funnel.

Some patients are additionally sold on overnight monitoring that does not actually occur. When a promise of postoperative observation resolves into rapid discharge to a hotel or recovery house, the patient enters the highest-risk postoperative hours under no clinical supervision. Postoperative arrangements, discharge disposition, and overnight monitoring are safety-relevant elements of office surgery. A patient cannot consent to overnight monitoring and receive a ride to a hotel.

The abandonment phase

The postoperative pattern at receiving hospitals is consistent. When a complication arises, and the patient contacts the original facility, the response, when it comes, often follows a recognizable sequence:

The Complication Playbook: A Recurring Postoperative Script

"I am out of town"; "Send the patient to the ER"; "That is expected swelling"; "The patient was noncompliant"; "Follow up Monday"; "This is not my complication"; "I no longer have privileges there"; "Have plastics see them."

When these responses recur across patients, facilities, and complications, they stop resembling isolated clinical judgment and start resembling a structured avoidance system. Postoperative care is not optional. It is part of surgery. A surgeon who performs an operation must have a credible plan for complications, real, named, reachable coverage.

A Sentinel Observation From the Bedside

The concern animating this editorial is not theoretical. In the author's clinical experience at a single receiving hospital, severe downstream complications after out-of-area cosmetic surgery have presented as both emergency-level resuscitation events and delayed wound-care referrals. This observation is not offered as epidemiologic proof or an incidence estimate. It is a sentinel bedside signal: clinicians at receiving hospitals are encountering complications that the state's current reporting architecture may not aggregate, trace, or analyze as part of a repeat-pattern facility problem. These observations were not derived from a systematic chart review and are presented only as hypothesis-generating clinical signals.

In recent clinical experience, some patients arrived with shock-level physiology after cosmetic surgery performed elsewhere; others sought wound-center care for significant postoperative wounds after similar out-of-area procedures. In de-identified emergency presentations, the receiving team attempted to create the simplest possible pathway for continuity by inviting the operating surgeon, or any covering surgeon, to evaluate the patient at the bedside. That bedside continuity did not occur. The problem was not that the surgeon was out of town. The problem was that the patient's care appeared to be out of ownership.

The receiving hospital can resuscitate, transfuse, intubate, debride, anticoagulate, admit to the ICU, and reconstruct. It cannot retroactively create the surgeon-patient relationship that should have existed before the incision. If a facility markets elective surgery to traveling patients, it must maintain real, named, reachable postoperative coverage. If it cannot, it has not built a surgical practice. It has built a transaction.

The point is not the number of cases observed by one physician. The point is that similar bedside signals cannot become public-health evidence unless Florida builds a reporting system capable of connecting downstream rescue care back to originating facilities.

The sentinel clinical observations are intentionally described in aggregate and category-level terms. Dates of service, patient ages, procedures, facilities, surgeons, locations, and other potentially identifying details have been omitted. The author will not disclose patient-level information outside legally authorized clinical, regulatory, or judicial processes.

Privatized Profit, Socialized Complications

The financial injury does not end with the patient. Many patients harmed by high-volume cosmetic surgery facilities are uninsured, underinsured, or unable to pay for the emergency care their complications require. The original procedure is paid up front. The complication arrives later through a different door: the emergency department. The facility that collected the surgical fee does not pay for the transfusion, ICU bed, vasopressors, antibiotics, debridement, skin grafts, wound vacs, home health, or repeat reconstruction. The business that sold the operation may have completed its transaction before the complication declares itself. The hospital, the public payer, the county system, the family, and the people of Florida inherit the rest.

The facility privatizes the profit. The public absorbs the complication: When a preventable complication from an elective cash-pay operation requires publicly funded emergency care, Florida residents are underwriting the risk of a private care-delivery model they did not choose and did not regulate adequately.

Why Civil Justice Often Fails These Patients

A patient may have devastating injury yet civil recovery may remain practically unavailable if defendants are uninsured, underinsured, asset-light, corporate-layered, dissolved, or renamed. Plaintiff attorneys evaluate not only negligence and damages but collectability. A meritorious case with no recoverable defendant may not be economically viable. This is especially harmful to lower-income and out-of-town patients who lack resources to pursue records, retain experts, or understand how ownership structures affect recovery. Section 458.320 establishes a financial-responsibility framework through insurance, escrow, or a letter of credit and, under specified conditions, permits physicians to practice without insurance subject to patient-notice and judgment-payment obligations [5]. That framework may be inadequate for a high-throughput elective surgery facility. The issue cannot be left to litigation alone.

The Core Reform: Mandatory Bidirectional Adverse-Event Reporting

Florida should create a mandatory adverse-event reporting system specifically for office-based cosmetic surgery complications. The current architecture captures isolated incidents but does not create a mechanism linking downstream rescue care back to the originating facility, surgeon of record, ownership structure, and payer burden [1]. That gap makes the pattern disappear.

Mandatory reporting should operate in two directions. First, the originating facility and surgeon of record should report any death, emergency transfer, hospital admission, ICU admission, transfusion, pulmonary embolism, fat embolism, sepsis, necrotizing infection, major wound loss, organ injury, or unplanned reoperation within a defined postoperative window [1]. Second, receiving hospitals, emergency departments, ICUs, wound centers, and treating surgeons should have a direct reporting pathway when they treat a major complication appearing related to recent office-based cosmetic surgery. Each report should identify the originating facility, surgeon of record, procedure type, anesthesia type, date of surgery, discharge destination, payer source, and clinical outcome. Florida's current reporting framework already recognizes that hospital transfer after office surgery is a reportable event; the receiving institution needs a corresponding pathway [1].

Mandatory reporting is the bridge between clinical suspicion and regulatory proof. Reports should be risk-adjusted and de-identified for public dashboards, but the data should not be hidden. Patients deserve to know which facilities generate repeated emergency transfers, ICU admissions, infections, hemorrhages, pulmonary emboli, and deaths. Florida cannot regulate a corridor it does not measure [1]. Repeat-pattern facilities should trigger automatic state review, inspection, corrective action, financial-responsibility review, and referral to appropriate licensing or enforcement agencies.

Additional reforms Florida can implement immediately

Mandatory Ownership Transparency

Florida should require disclosure of true facility ownership and management structure in state filings, patient-facing advertising, and consent documents. Patients should know who owns the facility, who controls scheduling volume, and who has authority to stop unsafe practices. Nominal physician ownership structures that obscure actual business control should be prohibited or penalized.

Volume-Triggered Financial Responsibility

The correct reform is not a universal malpractice mandate. Section 458.320 already provides a framework [5]. The reform needed is a volume-triggered financial responsibility requirement: once a cosmetic surgery facility exceeds defined thresholds, by case volume, procedure type, room count, or out-of-town marketing, it must maintain proof of professional liability coverage or equivalent state-approved financial responsibility as a condition of registration and continued operation. The requirement should extend to the surgeon of record, medical director, facility entity, and any management company exercising operational control. Facilities with prior sentinel events should face immediate enhanced-coverage requirements and automatic state audit.

Limits on simultaneous rooms

Florida should prohibit or strictly limit simultaneous cosmetic surgery rooms operating under one surgeon of record. The state should define, in enforceable regulatory language, what direct supervision requires and what concurrent room operation, if any, is permissible.

Credential transparency and procedure-specific privileging

Florida should require procedure-specific privileging before payment, travel, or consent for office-based cosmetic surgery. A physician should not be permitted to perform a cosmetic operation in an office-based facility unless an independent hospital or accredited ambulatory surgery center has credentialed that physician to perform the same or substantially similar procedure. Local peer credentialing is not perfect, but it is a meaningful external safeguard, independent of the cosmetic facility's financial incentives or advertising claims.

Procedure-specific privileging should apply not only to the surgeon of record but to every individual who performs a critical portion of the operation. No person should perform a critical surgical task in an office-based cosmetic surgery facility unless licensed and credentialed to do so. No surgeon should lend a license to rooms they cannot meaningfully supervise.

This editorial does not argue that only one specialty can perform cosmetic procedures safely. It argues that no physician should use an office-based facility to perform procedures they could not be credentialed to perform in a hospital or accredited ambulatory surgery center. If a physician's peers would not privilege that physician in a regulated institutional setting, the patient should not become the workaround. Credential transparency is an informed-consent issue. Procedure-specific privileging is a patient-safety issue.

Substantive Informed Consent

Patients should be told before surgery: whether their procedure is in a high-volume outpatient facility; whether the surgeon will cover overlapping rooms; whether the facility has a hospital transfer agreement; whether the surgeon holds active privileges at a licensed hospital or accredited ambulatory surgery center to perform the same or substantially similar procedure; and who will manage their complication if they have returned home. Consent should also disclose the physician's specialty training, certifying board, whether that board is ABMS-recognized, whether an independent institution has credentialed the physician for the same procedure, and whether postoperative coverage includes actual bedside availability or only telephone advice.

Whistleblower Protections and Public Dashboards

Nurses, anesthesia providers, and staff who report unsafe volume, ghost supervision, falsified documentation, or postoperative abandonment should receive explicit legal protection. Florida should create publicly accessible complication dashboards by facility and surgeon. Repeat-pattern facilities should be referred not only to licensing boards but also to the Attorney General, DOH, AHCA, and, where conduct warrants, law enforcement.

Conclusion

The I-95 complication corridor is not defined by geography alone. It is defined by a transfer of risk. The patient travels south for the promise. The complication travels north for the cleanup. Hospitals, reconstructive surgeons, emergency physicians, wound centers, public payers, families, and taxpayers absorb what the original transaction did not price honestly.

This is not an argument against cosmetic surgery. It is an argument for ownership and accountability. Responsible surgeons own their patients. Responsible facilities own their complications. Responsible regulators identify patterns before the next preventable death. The cheap operation is not cheap. It is underpriced at the point of sale and overpaid later by hospitals, families, public programs, and taxpayers. Florida should not wait for another patient to die on the drive home before treating this as a system failure.

A license cannot supervise three rooms at once. A consent form cannot replace postoperative care. And when no one owns the patient after the incision, the state owns the failure. The question before Florida's regulators, legislators, and medical boards is whether the state's existing data systems are capable of detecting this pattern before the next sentinel event [1,5].
